# Reflection grating fabrication for the Rockets for Extended-source X-ray Spectroscopy

**DOI:** 10.1007/s10686-025-10011-1

**Published:** 2025-06-14

**Authors:** Drew M. Miles, Ross McCurdy, Michael Labella, Randall L. McEntaffer, Fabien Grisé, Jake McCoy, James H. Tutt

**Affiliations:** 1 Physics, Math, and Astronomy, California Institute of Technology, 1200 E. California Blvd., Pasadena, CA 91125 USA; 2 Dept. of Astronomy & Astrophysics, Penn State University, 525 Davey Laboratory, 251 Pollock Road, University Park, PA 16802 USA; 3 Materials Research Institute, Penn State University, 491 Pollock Road, University Park, PA 16802 USA

**Keywords:** Reflection gratings, X-ray gratings, X-ray spectroscopy, Suborbital rockets, Electron beam lithography, Substrate conformal imprint lithography

## Abstract

The Rockets for Extended-source X-ray Spectroscopy (tREXS) grating spectrograph uses modules of reflection gratings to collect spectroscopic data from extended astronomical sources of soft X-rays. Two blazed master gratings were produced on silicon substrates with electron-beam lithography (EBL) and complementary nanofabrication processes that include KOH etching. Substrate-conformal imprint lithography (SCIL) was then used to create 191 replicas of the two grating masters for use in the flight instrument. Diffraction efficiency was measured for several replica gratings, which achieve a peak of $\varvec{>}$70% absolute efficiency near 0.22 keV and an average of $\varvec{\approx }$50% absolute efficiency across the measured band, from 0.18 – 0.8 keV. Here we detail the nanofabrication of the grating masters, including the EBL parameters and tREXS-specific fabrication considerations, and the SCIL replication process used to generate the final instrument gratings. A discussion of grating characterization and areas for future improvement is also presented.

## Introduction

The Rockets for Extended-source X-ray Spectroscopy (tREXS) grating spectrograph is a suborbital rocket instrument designed to collect spectroscopic data on extended astrophysical sources of soft X-rays. The tREXS instrument relies on sensitivity to line emission over a large field of view, $\approx$10 deg$^2$, across a soft-X-ray bandpass from $\approx$0.2 - 0.8 keV [17]. The observation targets are intrinsically faint – extended supernova remnants and enhanced regions in the soft-X-ray background – which makes grating diffraction efficiency and collecting area critical spectrograph parameters.

**Table 1:** The tREXS grating parameters, as designed, adapted from [18]

Grating parameter	Value
Avg. period *d*	181 nm
Blaze angle $\delta$	32$^\circ$
Blaze $n\lambda$	87.2 Å
Avg. $\gamma$	2.7$^\circ$
Avg. $\alpha$	42.2$^\circ$
Grating width	107 mm
Grating length	100 mm
Grating stack height	>145 mm
# gratings per stack	38

The design of the tREXS gratings is summarized here for context and is described more thoroughly in [17, 18]. The reflection gratings are implemented in an off-plane mounting that places the grating grooves nearly parallel to the direction of the incident X-rays. The X-rays meet the grating surface at a grazing incidence angle and then diffract according to

1 $$\begin{aligned} sin(\beta ) = \frac{n\lambda }{dsin(\gamma )} - sin(\alpha ), \end{aligned}$$

where $\alpha$ and $\beta$ are the azimuthal angles of the incident and diffracted ray, respectively, *n* is the diffraction order number, $\lambda$ the wavelength, *d* the spacing of the grating grooves, and $\gamma$ the half-cone opening angle between the incident ray and the grating groove direction [2]. By shaping the groove facets to a specified angle relative to the grating surface plane (i.e. producing a blazed grating), incident light will preferentially diffract to a particular order $n_b$ for a given wavelength, $\lambda _b$:

2 $$\begin{aligned} n_b\lambda _b = dsin(\gamma )[sin(2\delta - \alpha ) + sin(\alpha )], \end{aligned}$$

where $\delta$ is the blaze angle of the groove facets. In designing the grating parameters for an instrument, the grating groove spacing, blaze angle, and orientation of the grating structure relative to the incoming beam can be optimized for a set of $n_b\lambda _b$ based on the expected spectral features from the observation targets.

Table 1 summarizes the parameters to which the tREXS gratings were designed. The gratings have a variable line spacing along the groove direction, with the groove spacing changing every 1 mm to produce 100 segments of constant density. The period converges from 185.7 nm to 176.3 nm in the 1-mm increments at a rate that matches the overall convergence angle of the instrument’s optics. The blaze angle and grating orientation relative to the telescope beam were determined based on optimizing throughput at key transitions in the soft-X-ray band, particularly for 4$^{th}$-order O VII  at $n\lambda$ = 87.2Å. The physical dimensions of the gratings were driven by the size of the instrument’s telescope beam that the gratings intercept; at the position of the gratings in the instrument layout, 50-mm forward of the optics, the converging telescope beam is $\approx$107-mm wide and $\approx$145-mm tall [17]. The 100-mm grating length represents the largest physical footprint achievable on the 150-mm-diameter wafers used for grating fabrication.

## Methods

The tREXS spectrograph requires two unique grating designs and 152 total gratings. Two “master” gratings were manufactured using electron-beam lithography (EBL) and KOH etching, and nearly 200 replicas were produced using substrate-conformal imprint lithography (SCIL, [19, 21–23]) to populate the flight grating modules. This section provides an overview of the grating fabrication processes, including EBL exposure settings, fogging mitigation, post-EBL processing, and grating replication.

### Master grating fabrication

**Fig. 1:**
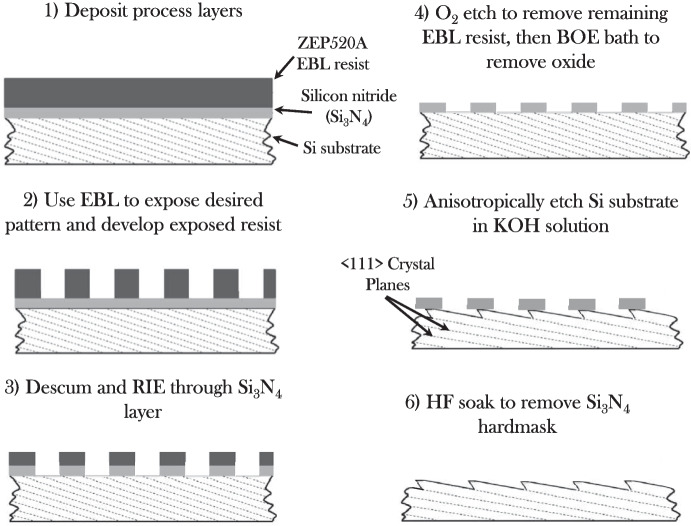
A process diagram for the master grating fabrication using EBL and KOH, adapted from [5] and [7]. In Step 1, a layer of Si$_3$N$_4$ and an EBL resist, ZEP520A, are deposited on a Si substrate. In Step 2 the grating pattern is exposed with the EBL tool and developed to leave behind the specified groove layout in the resist. In Step 3, the grating pattern is transferred through the Si$_3$N$_4$ layer to the surface of the Si wafer. The residual EBL resist is stripped in Step 4, and the KOH etches down to the $\{$111$\}$ crystal planes, represented by the dashed lines in the substrate, in Step 5. Finally, the KOH etch is halted and a HF acid soak is used to remove all remaining Si$_3$N$_4$ in Step 6

The master grating fabrication generally followed the approach described in [16] and summarized in Fig. 1. Fabrication begins with the acquisition of a monocrystalline Si substrate wafer cut to the orientation required for KOH etching to produce blazed facets of the required angle. A 30-nm layer of Si$_3$N$_4$ is deposited via low-pressure chemical vapor deposition to be used as a hardmask during KOH etching, and the wafer is coated with an EBL resist, $\approx$150 nm of ZEP520A in this work [24]. Following process optimization, the specified grating pattern is exposed into the resist and developed, leaving behind a binary groove layout as depicted in Step 2. All EBL processing in this work was performed with a 100-kV Raith EBPG 5200 [20] at the Penn State University Nanofabrication Laboratory.

The EBL-defined groove layout is then transferred through the Si$_3$N$_4$ layer to the surface of the Si substrate with a reactive-ion etch (RIE). An oxygen-based RIE is then used to remove the remaining resist so that the EBL-defined groove layout is retained only in the Si$_3$N$_4$ mask. Following a short buffered-oxide etch (BOE) to strip the native Si oxide layer, the Si wafer is submerged in a KOH bath to etch the Si crystal planes. Once the desired blazed facet dimensions are achieved, KOH etching is halted with a DI water rinse and the wafer is submerged in a hydrofluoric acid (HF) bath to strip the remaining Si$_3$N$_4$. At the conclusion of this processing, a grating is produced with a blaze angle set by the orientation of the Si crystal planes and a groove layout determined by the EBL exposure.

**Fig. 2:**
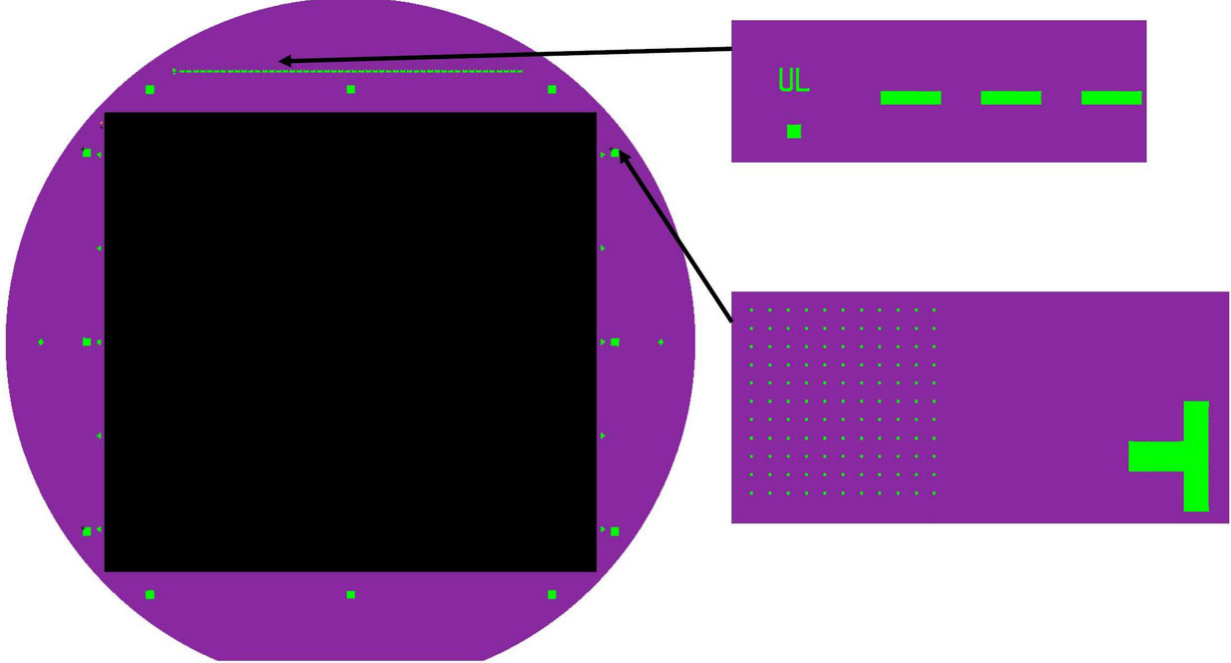
A depiction of the EBL exposure regions for the tREXS master grating pattern (dark rectangle), EBL alignment markers (array of small squares in the expanded view at image right), a dashed line of markers for coarse alignment along the top edge of the grating, and dicing alignment markers to trace the outline of the grating pattern for post-fabrication processing (rotated “T” in expanded view at image right). All patterns are shown on a 150-mm-diameter wafer represented in purple [13]

Prior to the production of the flight grating pattern, several processes were completed to prepare the wafers. First, a series of crystal alignment markers were exposed following Steps 1 - 5 in Fig. 1. Alignment of the EBL tool to the crystal planes is critical to ensure that the EBL-exposed grating pattern is well aligned to the {111} planes in the Si wafer to produce the smooth, angled facets required for highly efficient diffraction. A series of small, $\approx$20-$\mu$m, gratings with 3.75-$\mu$m periodicity were exposed in an arc near the top and bottom edges of the wafer at 0.5$^\circ$ intervals. The fully processed alignment gratings were then imaged with a field-emission scanning-electron microscope (FESEM) to identify the angle at which the gratings are aligned to the Si crystal planes. The Si wafer was then cleaned and prepared with an additional set of alignment markers to align the previously measured crystal orientation to the EBL tool coordinate system. These EBL alignment markers, 20-$\mu$m squares arrayed in 11 $\times$ 11 grids with 150-$\mu$m pitch, are arranged around the perimeter of the grating footprint as depicted in Fig. 2 and are referenced throughout the EBL exposure to regularly align the tool coordinate system to the grating pattern.

After the full implementation of the crystal and EBL alignment markers, the substrate wafer was prepared for the grating pattern exposure. Following a cleaning step but prior to the deposition of the new layer of ZEP520A, the wafer was submerged in SurPass 3000 [3] to promote adhesion between the EBL resist and nitride coating. A 150-nm layer of ZEP520A, mixed 1:1 with anisole, was then deposited onto the wafer and the full grating pattern was exposed over a continuous 156.6-hour period following an 8-hour pre-exposure settling period for the wafer in the EBL tool. The EBL-exposed resist was then developed in n-amyl acetate for 180 seconds and submerged in isopropyl alcohol for 120 seconds to quench the developer, after which the wafer was dried with manual application of a gaseous nitrogen gun.

#### EBL exposure settings

One challenge of the parallel-groove segmentation used in this work is that uniform EBL exposure settings used across the full grating will result in a variable groove width as the groove spacing changes; as the periodicity converges, the ratio of unexposed:exposed resist will vary if the exposure parameters are held constant. For a target post-etch groove plateau width of $\approx$30 nm (refer to Section 2.1.3), the $\approx$10 nm variation in groove spacing across the grating surface and subsequent variation in etch-mask tab widths can lead to etch inconsistencies (e.g. over etching or under etching in certain segments and the risk of completely undercutting the Si$_3$N$_4$ mask) and nonuniformities in the final grating pattern. To avoid such etch-induced grating nonuniformities, the width of the EBL-exposed grooves were tuned in each 1-mm-long segment of the grating such that the width of the unexposed regions was conserved even as the groove spacing changes.

The variable groove spacing was compensated for in the EBL settings by applying a bias to the specified exposure area for each different segment to tune the size of each exposure region. With this approach, a single set of beam parameters (electron dose, beam diameter, etc.) can be used with a variable bias value to produce consistently sized groove features despite the variable groove spacing from segment to segment. For the tREXS masters, a 40 nA beam current and 400 $\mu$m beam aperture were used to produce a 30 nm beam spot size, $\approx$1.5$\times$ the 20-nm beam step size to provide uniform exposure over the full groove width.

Table 2 summarizes many of the key parameters used in the EBL exposures for the master gratings. This work used GenISys BEAMER software [8] to generate a custom, variable writing grid to best match the grating design and optimize the total EBL exposure duration. The write grid resolution, which specifies resolution of the EBL grid onto which the specified pattern is mapped, was set to $d_i$/100, where $d_i$ is the groove spacing for each segment *i*. Similarly, the beam step size, the physical displacement between individual beam shots, was set to $d_i$/10.

**Table 2:** EBL exposure parameters used for the tREXS master gratings

EBL parameter	Value
Accelerating voltage	100 kV
Beam current	40 nA
Beam aperture	400 $\mu$m
Beam spot size	30 nm
Electron dose	200 $\mu$C/cm$^2$
Write grid resolution [nm]	$d_i$ / 100
Beam step size [nm]	$d_i$ / 10
Mainfield size	200 $\mu$m $\times$ 200 $\mu$m
Subfield size	4.52 $\mu$m $\times$ 4.52 $\mu$m
Fracture mode	LRFT
Field ordering, main and sub	Fixed, MeanderX
Overlap, mainfields [nm]	100
Overlap, subfields [nm]	160

Some parameters were tuned based on each grating segment’s groove spacing, $d_i$, to produce a constant groove width regardless of the groove spacing

The subfields were set to the maximum size allowed by the Raith EBPG 5200 tool to minimize the exposure time without a loss in pattern fidelity: 4.52 $\mu$m. The maximum allowable mainfield size, 1 $\times$ 1 mm, was determined to be too large to retain the exposure quality desired for tREXS; a larger mainfield presents a larger area over which the mainfield calibration must hold, and regions near the perimeter of the maximum field size may deviate from the nominal exposure settings near the center of the fields. To balance exposure quality with write speed (smaller mainfields requires more total stage movements and a longer total write time), 200 $\mu$m mainfields were used.

The fracture mode used for tREXS was Large Rectangle Fine Trapezoid (LRFT), which divides input patterns into exposable rectangular shapes and trapezoids, where necessary. The EBL settings further used fixed field ordering and a MeanderX traversal type for both main and subfields. A 100-nm and 160-nm overlap region was specified for mainfields and subfields, respectively, for both the *X* and *Y* directions to define the size of the area in which the tool can overlap the neighboring write fields. The overlap regions allow the tool to complete a specific write shape before proceeding to the next field, ensuring groove continuity across field boundaries.

#### EBL fogging mitigation

Fogging is a known effect in EBL processing that results from electrons scattering from the target substrate back toward the instrument’s objective lens. The electrons that scatter again back toward the EBL resist produce the fogging effect: a higher electron dose in the affected region and distortions in the exposed features [1, 11]. For many small EBL exposures, including the developmental pieces for the tREXS gratings, the fogging effect is negligible and was not detected in any of the pre-master development work. For the master exposure, which includes a write area >100 cm$^2$, however, the additional electron dose from the fogging effect can alter the grating grooves and contribute to overexposed and collapsed grooves.

To mitigate effects from fogging, an experimental approach was used to generate a spatially dependent bias map for the electron dose used in the master EBL exposures. First, arrays of eighteen 1-mm$^2$ segments of the tREXS pattern were spaced at $\approx$20 mm intervals and exposed with a background grating pattern approximating the total write area and electron dose of the full master but with different groove spacing and feature sizes. The background grating simulated the electron dose (and therefore induced fogging effect) of the full master pattern but the larger groove spacing allowed for a reduced write time from the >100 hr required for the full master exposure. Each set of 18 grating segments included a range of electron doses and bias values to observe the fogging effect with a range of beam parameters. The test exposures, which took $\approx$10% of the write time of the full master, were then developed and imaged with a FESEM. An interpolated bias map was then produced to determine the necessary bias applied to the notional exposure width used to define each groove in order to achieve the desired feature size as a function of position in the exposed area.

The test exposures demonstrated that at an electron dose of 200 uC/cm$^2$ minimized fogging effects and allowed for a sufficiently high dose to clear the resist. The bias map was then applied with a standard dose of 200 uC/cm$^2$ and a one-dimensional interpolated variable bias that varied along the 1-mm segments in the groove direction. A master grating pattern was prepared using the process steps described in the previous section (crystal alignment markers, EBL alignment markers, SurPass 3000, and full grating exposure) and with the optimized electron dose and bias variation. The groove fidelity was confirmed after developing the exposed resist, the pattern was subsequently transferred through the Si$_3$N$_4$ layer and the remaining resist was stripped as outlined in Steps 3 - 4 of Fig. 1. The exposed groove pattern was deemed to meet tREXS requirements for the first master grating, M1. The process was repeated for the second master, M2, which carries the same groove layout rotated 180$^\circ$ on the substrate wafer in order to produce a grating with the opposite blaze direction as required by the instrument design.

**Fig. 3:**
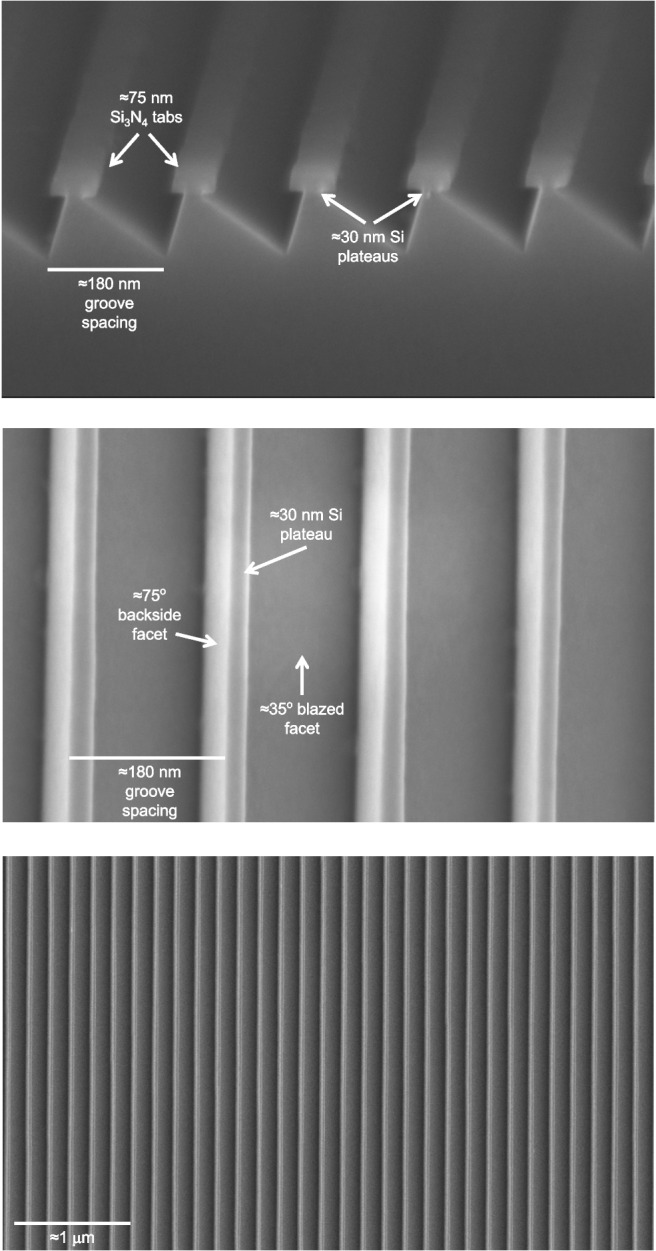
Top: A cross-sectional FESEM image of a KOH-etched tREXS development grating. The KOH etch has undercut the $\approx$75-nm initial Si$_3$N$_4$ tabs to produce blazed facets with $\approx$30-nm plateaus at the groove apices. Middle: A top-down FESEM image of tREXS M1 showing the blazed facets, narrow Si plateaus, and steep facet backsides. Bottom: A top-down FESEM image of M2 showing the uniformity over a larger region

**Fig. 4:**
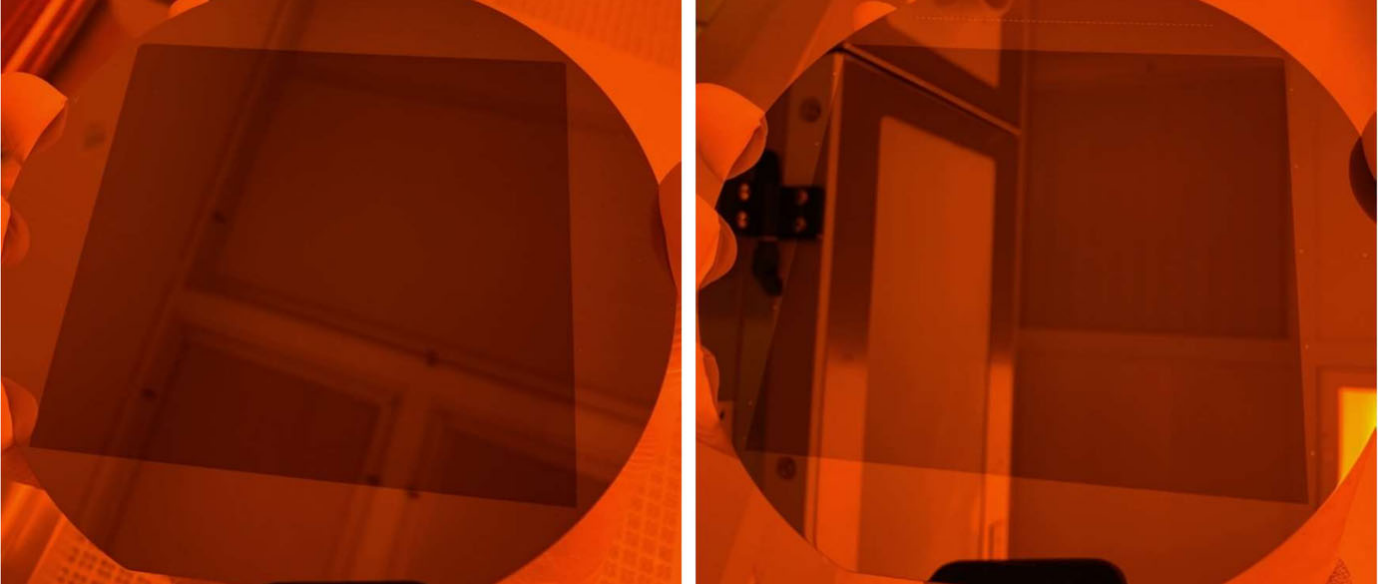
Images of master grating M1 (left) and M2 (right) after all fabrication steps are completed. The rectangles of darker contrast are the active grating patterns etched into the Si wafers [13]

**Fig. 5:**
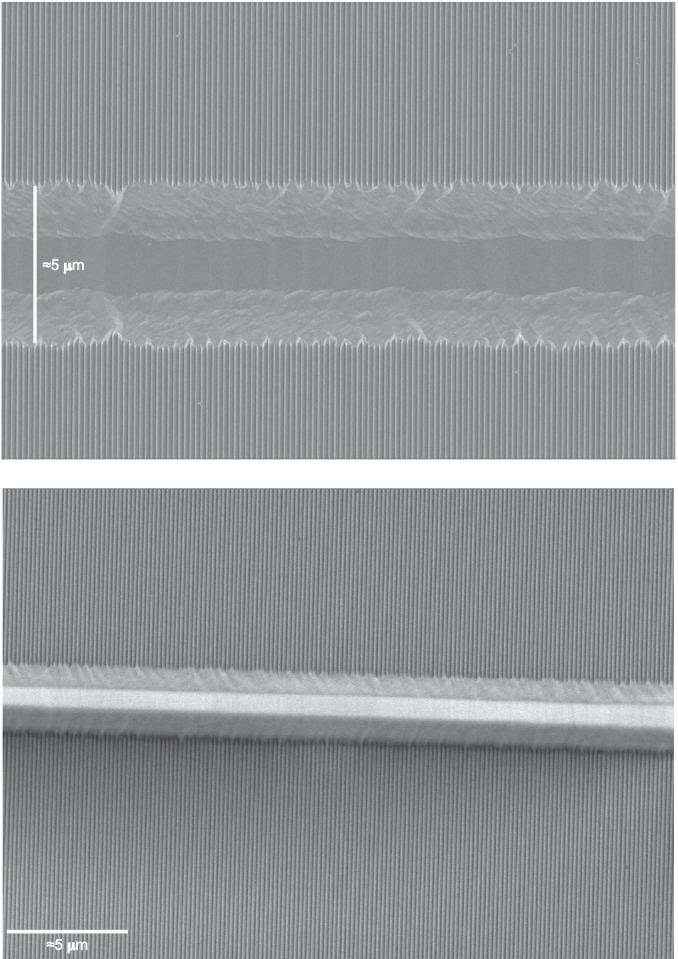
Top: a top-down FESEM image of a tREXS master grating after full KOH processing. Regular grating grooves are visible around a much larger etch trench, the result of inconsistent groove spacing at a segment boundary. Bottom: A top-down FESEM image of a grating replica in cured sol-gel resist on a fused silica wafer. A relatively large, $\approx$5 $\mu$m tall and $\approx$3 $\mu$m wide, plateau that was inverted from the etch trench is visible above the grating grooves (image credit: SCIL Nanoimprint Solutions)

#### KOH processing

As indicated in Fig. 1, the EBL-defined groove layout is transferred through the Si$_3$N$_4$ layer directly to the surface of the wafer via RIE. Once the resist is stripped, the grating pattern is retained in the residual Si$_3$N$_4$ “tabs”. The objective for the KOH etching was to produce the blazed facets defined by the orientation of the $\{$111$\}$ planes and leave behind a groove plateau that was $\lessapprox$30 nm wide; small enough to be completely shadowed by the groove height in the flight grating configuration (refer to Section 2.2) but large enough to avoid any effects from over etching.

Prior to the KOH etch, the grating was first submerged for 10 s in a BOE bath consisting of a 10:1 ammonium fluoride (NH$_4$):HF mixture to strip the native oxide layer. After a DI water rinse to neutralize the BOE etch, the full wafer was submerged in a 45% KOH bath. For the master gratings, a room temperature KOH bath was used to allow control over the etch rate without requiring prohibitively long KOH exposures. The two master gratings were submerged in the KOH bath and removed every 30 minutes to measure the etch evolution with FESEM imaging. In the end, M1 was etched for 120 minutes and M2 for 90 minutes, with the difference due to the fact that M2 began with smaller initial Si$_3$N$_4$ tabs after its EBL exposure. Finally, a post-KOH DI water rinse and 10-minute soak in 49% HF halted the etching and stripped the Si$_3$N$_4$, leaving the blazed gratings etched completely into the Si substrates. A cross-sectional FESEM image of a development sample is shown in the top panel of Fig. 3, depicting a $\approx$75-nm initial Si$_3$N$_4$ tab and 30-nm residual Si plateau undercut beneath the Si$_3$N$_4$, and top-down FESEM images of M1 and M2 are in the middle and bottom panel of Fig. 3, respectively. Figure 4 shows the two master gratings after all fabrication processing.

The KOH processing also revealed a defect resulting from the change in groove spacing at the segment boundaries. The change in groove spacing (and therefore groove placement) at each boundary created a periodic, grating-like structure with a spacing equal to the segment size in the cross-groove direction of the primary grating. For tREXS, designed for a bandpass from $\approx$15 - 50 Å, the impact of the defect-induced grating structure with 1-mm groove period on X-ray diffraction was not a principal concern. An additional effect was the size of the KOH-etched trench; the discontinuity at the segment boundaries resulted in a significant over etch and subsequent trench in the cross-groove direction. An example of one such defect is visible in the top panel of Fig. 5. These $\approx$5-$\mu$m wide trenches reduced the effective surface area of the gratings by $\lessapprox$1% and were not considered to invalidate the gratings for use in the instrument.

**Fig. 6:**
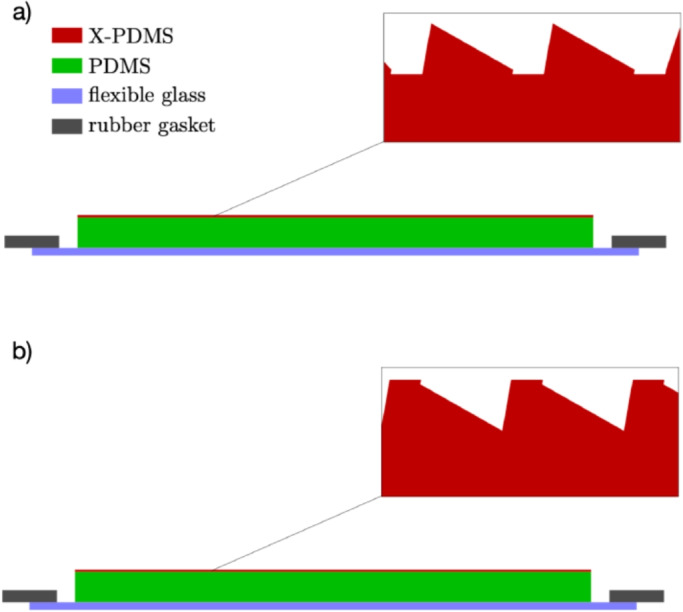
The SCIL stamp-making process, adapted from [19] and [23]. In a), the master template is coated with a thin layer of X-PDMS and a mold is produced by pressing a thick layer of PDMS and a flexible glass substrate against the master template. The X-PDMS layer creates a stamp with an inverted groove pattern relative to the master (refer to the top panel of Fig. 3). In b), the process is repeated to create a second X-PDMS stamp from the first stamp, which inverts the pattern again back to the groove profile of the original grating. The second stamp is then used to create replica gratings with an inverted groove profile relative to the master, similar to the first stamp’s profile depicted in the inset of the top panel

### Grating replication and post-replication processing

The resources required to produce grating masters make it prohibitive to fabricate a large number of gratings using EBL and KOH etching alone. To realize the 152 flight gratings (76 each of M1 and M2) required for tREXS, the two master gratings were replicated dozens of times each to produce copies that are then integrated into the instrument. SCIL was used for the grating replication due to its ability to precisely replicate grating features over a large area and to produce many replicas from a single master grating [22, 23]. With SCIL, flexible composite “stamps” are produced from the master template and are then used to make replicates on additional substrates. The masters are cleaned and deposited with an anti-stiction layer to allow the imprint mold stamps to release easily from the grating surface. The stamp-making process includes a 500-$\mu$m layer of polydimethylsiloxane (PDMS) between a 200-$\mu$m-thick, 200-mm-diameter flexible glass sheet and the master template, which is coated with a 50-$\mu$m layer of X-PDMS, a modified, high-modulus PDMS developed at SCIL Nanoimprint Solutions [23]. This stamp can then be imprinted into a resist on a new wafer to produce grating replicas.

A flexible X-PDMS stamp that is molded from a master grating carries an inverted grating pattern relative to the master; the stamp adheres to the surface of the master grating such that the base of each groove on the master grating becomes the groove apex on the stamp. Grating replicas made from the initial X-PDMS stamp repeat this inversion process to imprint the grating pattern from the stamp into a resist on the replica substrate, resulting in a replicated grating that carries the same groove profile as the original master. For tREXS, the gratings were designed to have the master grating pattern inverted so that the $\approx$30-nm plateau at the apex of each groove (visible in Fig. 3) is instead imprinted as the groove trough, creating sharp blazed facets that shadow the troughs when oriented correctly relative to the incident light. A second SCIL mold was therefore produced to create an X-PDMS stamp that carries the original grating pattern and produces replicas with the inverted pattern. Figure 6 depicts the stamp-making process, including insets showing the groove profile of the first stamp and second stamp in Fig. 6a and b, respectively. The second stamp was then imprinted into a $\approx$100 nm layer of NanoGlass sol-gel resist deposited on the replica substrates, 425-$\mu$m-thick fused silica wafers. Using an automated SCIL (AutoSCIL) tool, the replication process from the X-PDMS stamp can achieve yields of >40 replicas per hour [22].

During the replication process, the impact of the segment boundary over etch from the master grating fabrication was exacerbated: the conformality of the imprint process allowed the X-PDMS to fill the etch trenches, resulting in corresponding raised features on the first stamp that carry through the rest of the SCIL replication process. These raised features have a height of $\approx$5 $\mu$m, $\approx$100$\times$ taller than the $\approx$60-nm grating groove depth, and occult a portion of the grazing-incidence light from the telescope beam and reduce the effective collecting area of each grating by $\approx$10%. A top-down FESEM image of one such defect is shown in the bottom panel of Fig. 5. Further, the raised defect contributed to imperfect conformality during the replication process and required iteration with stamp production and resist parameters to ensure high-quality imprints. In addition, each sol-gel imprint was cured at 150$^\circ$C, a higher temperature than was planned in grating design. The discrepancy resulted in a larger-than-expected amount of shrinkage in the replica gratings and a reduced blaze angle from the notional design.

After 9 replica substrates were consumed for process development, 191 total gratings were produced from the SCIL processing, roughly split between replicas from M1 and M2. In addition to fulfilling the required number for the flight instrument, the 191 gratings replicated for tREXS represents the most X-ray gratings generated using the AutoSCIL process, a key technology for future, large X-ray missions that may require thousands of identical gratings.

Following SCIL processing, the replicated gratings were coated with Ni. A coating thickness of $\approx$15 nm was deposited using a High Uniformity Lift-off Assembly (HULA) fixture with a Temescal F-2000 [6] electron-beam evaporation system at the Penn State University Nanofabrication Laboratory. The final processing performed on each grating was to dice the grating pattern out of the fused silica wafers used for SCIL processing with a DISCO DFD6240 automated dicing saw at the Penn State University Materials Research Institute. Though the actual extent of the grating pattern was 100 mm $\times$ 107 mm, the diced regions were 100 mm $\times$ 109 mm to allow for an extra 1 mm on each side of the grating width to hold alignment apparatus when the gratings were integrated into the instrument [13].

## Results

### Atomic force microscopy

**Fig. 7:**
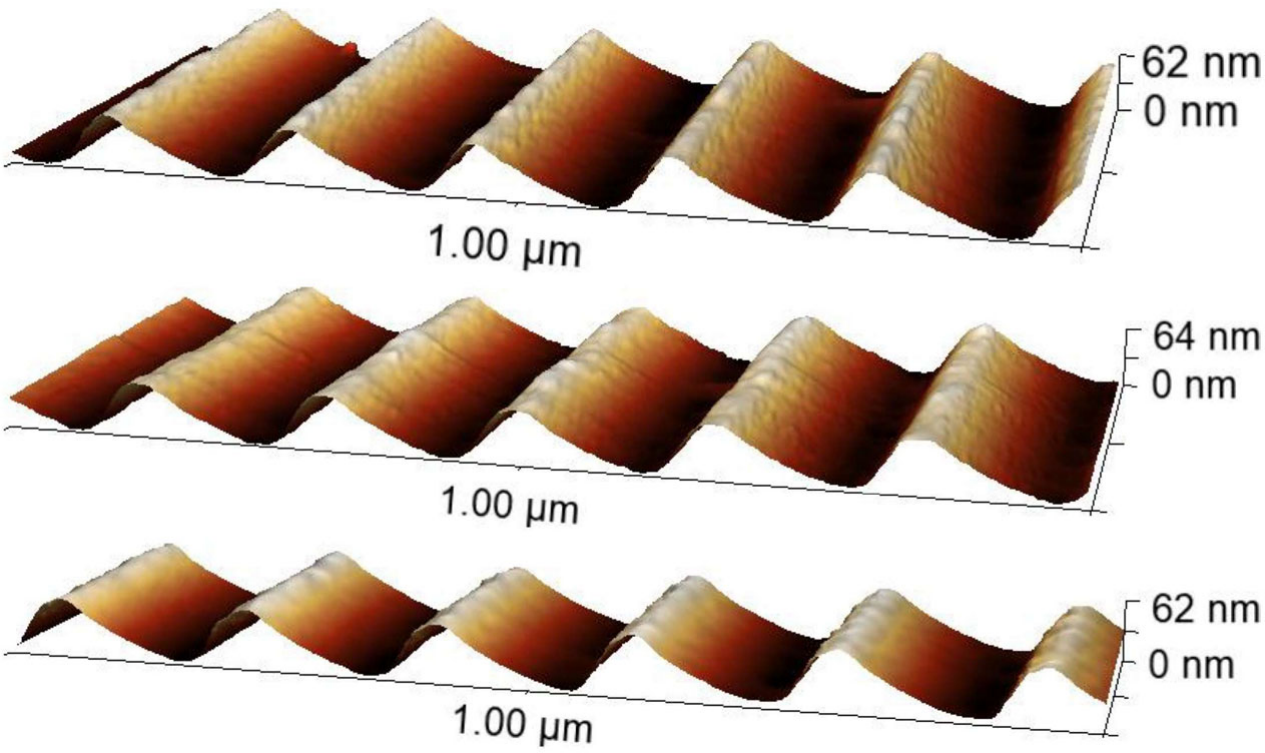
AFM images from the center of three tREXS replicas. The top two scans were from replicas from master grating M1, and the bottom scan is from a replica from M2. The M2 replica image was rotated 180 degrees to orient the blaze profile in the same direction as the M1 replicas

After all manufacturing processes and prior to being implemented into the instrument, a subset of the replica gratings were imaged with atomic force microscopy (AFM) to assess their surface profile and measured for diffraction efficiency. AFM scans were collected near the top, center, and bottom of each grating pattern. The replica groove profiles, a sample of which are shown in Fig. 7, were found to have rms surface roughness measured with AFM of $\approx$0.5 – 1.5 nm after the deposition of the Ni coating. Estimates of the blaze angle were made by scanning 60-nm-wide regions along the facet face for several grooves in several different regions of the grating. The average measured blaze angle was $\delta ~\approx$ 28$^{+1.5}_{-4.0}$ degrees, resulting in a reduction from the master gratings of $\approx$7$^\circ$ compared to the projected shrinkage of $\Delta \delta ~\approx$ 2$^\circ$ based on the findings in [19]. It was determined that the cause of the greater facet angle shrinkage was due to the temperature used to cure the sol-gel resist, 150$^\circ$C in this work compared to $\approx$50$^\circ$C - 90$^\circ$C in prior work.

The shallower blaze angle impacts the tREXS spectrograph performance by shifting the peak of the blaze envelope defined by $n_b\lambda _b$ in (2), concentrating light into different orders than the nominal design for a given wavelength. For O VII, for example, the blaze order shifts from $n~=~4$ to $n~\approx$ 3.5. To compensate for this effect, the incidence angle $\gamma$ was modified from the designed 2.7$^\circ$ to 3.0$^\circ$ and $\alpha$ was increased from 42.2$^\circ$ to 47.3$^\circ$. Though the steeper incidence angle results in a lower average diffraction efficiency, the change in configuration, which is accomplished with the way the gratings are aligned into stacks and integrated into the instrument, allowed for the best balance of throughput losses and dispersion considerations for the shallower blaze angle, as guided by instrument models. The updated configuration places the blaze order for O VII at $n~=~3.8$.

### Diffraction efficiency

Three replica gratings were also measured for diffraction efficiency at Beamline 6.3.2 at the Advanced Light Source at Lawrence Berkeley National Laboratory [9]. Each grating was installed in the beamline endstation in at a grazing incidence angle representative of the implementation in the instrument following the procedures described in [16] and [19]. After measuring the orientation of the grating relative to the X-ray beam delivered by the beamline monochromator, diffraction efficiency data were collected at a range of energies from 180 - 800 eV by comparing the intensity of each diffracted order to the intensity of the direct monochromator beam at each energy.

**Fig. 8:**
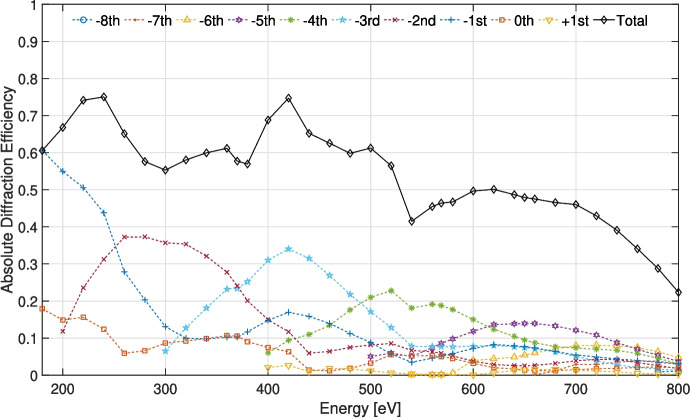
Absolute diffraction efficiency as a function of energy for the M1R1 tREXS replica grating. The solid curve shows the total efficiency in all diffraction orders (not including 0$^{th}$ order), and each dashed curve shows an individual diffraction order. Measurement errors are smaller than the plotted data markers

**Fig. 9:**
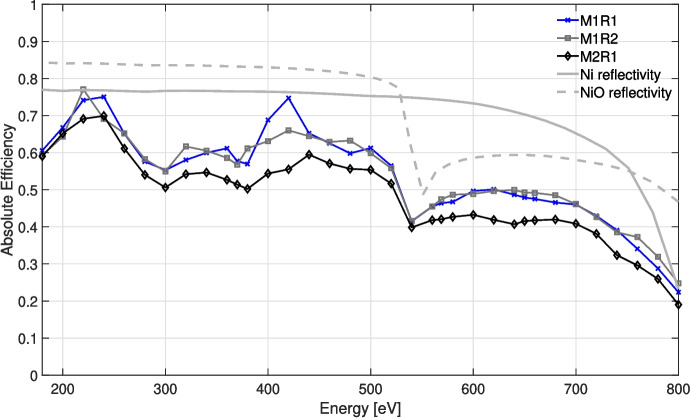
The total absolute diffraction efficiency (not including 0$^{th}$ order) of three measured tREXS replica gratings, along with the reflectivity of a Ni coating and a NiO coating. M1R1 and M1R2 are two replicas from master grating M1, and M2R1 is a replica made from M2. The reflectivities were collected from the CXRO X-ray Database for a thick coating with 1 nm rms surface roughness and an incidence angle $\gamma$ = 2.95$^\circ$. Measurement errors are smaller than the plotted data markers

Figure 8 shows the absolute diffraction efficiency of a tREXS M1 replica, M1R1. The solid curve shows the total diffraction efficiency – the sum of all orders excluding 0$^{th}$ order – across the measured band, and each dashed curve shows contributions from individual diffraction orders. The mean total diffraction efficiency measured was $\approx$54% from 180 - 800 eV, with a peak total absolute efficiency of $\approx$75% at 240 eV and 420 eV. Single-order contributions to the total efficiency are dominated by the blaze orders at lower energies, with the blaze response less dominant above $\approx$600 eV, possibly due to replication- and coating-induced facet rounding at the apex of each groove. Figure 9 shows the total absolute diffraction efficiency for each of the three gratings tested: two replicas from M1 (M1R1 and M1R2), and one replica from M2 (M2R1). We attribute the small deviations in measured efficiency for the two M1 replicas to variations in test configuration, where the experimental $\gamma$ was 2.95 ± 0.15$^{\circ }$ in each case, and the efficiency differences from M1 replicas to the M2 replicas to a combination of test configuration and potential differences in fabrication and replication of the different gratings.

The measured efficiency falls short of the performance achieved with previous replicas manufactured with similar processes [15]. In addition to the impacts to the facet shape from the replication process and the lost collecting area due to the segment boundary defects, the decreased diffraction efficiency above $\approx$500 eV may be due to the reflective coating. Figure 9 shows reflectivity curves for a Ni coating and NiO coating, with the reflectivity data collected from the Center for X-ray Optics (CXRO, [12]) X-ray database for reflectivity of a thick mirror with 1 nm rms surface roughness at an incidence angle of 2.95$^\circ$, the approximate measured angle at which the replica was tested. The efficiency curves more closely follows the NiO reflectivity, particularly near the O-K edge near 530 eV and due to absorption from the Ni-L shell above 800 eV. This result indicates that the deposition of the Ni coating may have resulted in oxidation that influences the diffraction efficiency.

To further investigate the potential NiO contributions to grating performance, a coated replica was examined using X-ray Photoelectron Spectroscopy. The resulting measurements indicate the presence of oxygen in the first $\approx$3 nm of the Ni coating, including $\approx$65% oxygen content at the coating surface. After $\approx$3 nm, the coating is consistent with Ni until the interface with the Si substrate. Though only a small fraction of the total coating thickness, the NiO layer has a significant impact on incident X-rays; the attenuation depth for soft X-rays in NiO at an incidence angle of 3$^\circ$ is $\approx$3 nm across much of the tREXS bandpass (from CXRO’s X-ray database), indicating that the NiO layer can have a substantial impact on the efficiency performance consistent with the measurements in Figs. 8 and 9.

**Fig. 10:**
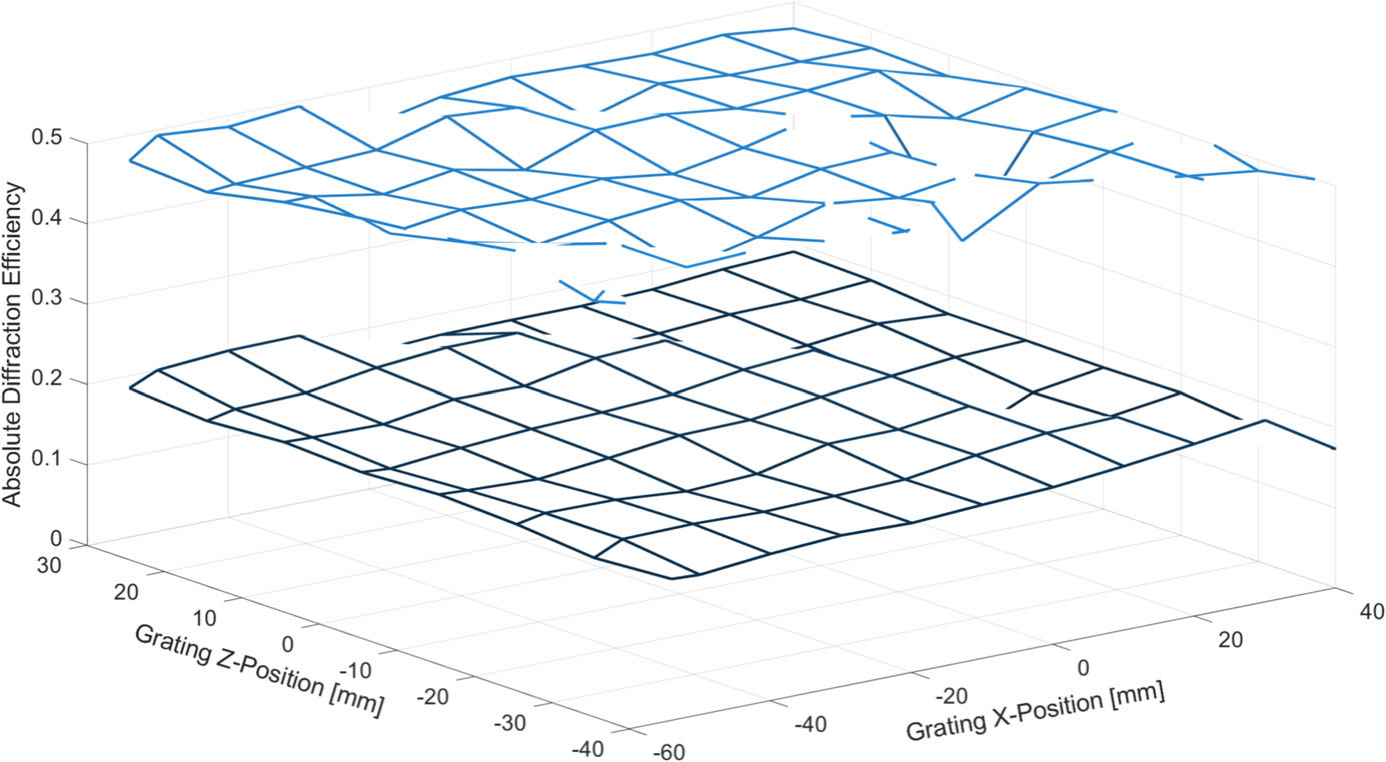
Diffraction efficiency at 568 eV as a function of position on the grating surface for a tREXS replica grating. Measurements were taken at several positions in the cross-groove (*x*) and groove (*z*) directions. The lower data set is absolute efficiency in 4$^{th}$ order and the higher set is the total absolute diffraction efficiency at this fixed energy

In addition to the measurements at the range of energies at a single point on each grating, data were collected at a fixed energy across the surface of a grating. The absolute diffraction efficiency at 568 eV, the energy of the resonance transition in O VII , is plotted in Fig. 10. The total diffraction efficiency (sum of diffracted orders excluding 0$^{th}$ order) and the efficiency of 4$^{th}$ order, the nearest integer order to the blaze order $n_b$ at that energy for the tREXS design, were measured at 10-mm intervals across the full stage travel allowable by the beamstation’s sample staging, resulting in 90 measurement points across the grating surface. The mean efficiency and standard deviation of measured points across the surface is 48.9 ± 2.2% for the sum of orders and 18.7 ± 1.6% for 4$^{th}$ order.

## Discussion

The challenges encountered in this work have resulted in several lessons learned that can help improve the performance of future gratings fabricated with the methodology used here. First, though a simplified approximation to a constantly varying line spacing was used for ease of fabrication and the relatively low resolving power requirements for tREXS, the discrete period jumps at the segment boundaries complicated both the master fabrication and the replication. A continuously varying groove density such as those designed for higher-resolution spectrographs (e.g. [4] and [14]) would have avoided the segment boundaries and the resulting performance losses due to the etch trenches. Alternatively, a boundary layer between each segment could be used to separate regions of changing densities and as a KOH mask to avoid etch trenches, albeit at the cost of a small loss in collecting area ($\approx$1% for a $\approx$10-$\mu$m layer in the tREXS design, for example).

The effects of EBL fogging could also be lessened with modifications to the master fabrication approach. Using a thinner EBL resist layer would allow for a lower required electron dose to fully expose the resist in a given region, which would in turn lessen the impact of EBL fogging. A thinner resist layer would also help with mitigating groove collapse; the aspect ratio (ratio of the groove width to groove height) for a grating would increase for a set exposure width as the resist thickness decreases. Finally, master grating production using a negative-tone EBL resist could further reduce the risk of EBL fogging by decreasing the overall area exposed for a grating design that targets groove widths that are less than half of the groove period, as was the case for the tREXS design.

In addition to the replication challenges attributed to the KOH etch defects, which contribute both to effective area loss due to raised structures in the replica gratings and to challenges with conformality in the imprint process, greater control over the cure temperature would have improved the efficiency performance in the designed configuration. While this work’s instrument design assumed comparable imprint shrinkage to previous studies, a higher temperature was used for the these replicas, which led to increased shrinkage and a shallower grating blaze angle. The effect was partially mitigated by modifying the configuration in which the gratings were installed in the instrument, but efficiency losses were realized due to both an increased incidence angle in the revised configuration and a modified blaze envelope $n_b\lambda _b$. Ensuring that the design specifications properly account for the realized fabrication processes can minimize the impact of production inconsistencies.

Finally, a more thorough analysis of coating methods and materials can help maximize the overall diffraction efficiency. Though this work included a comparison of different deposition techniques, the comparative analysis was limited to AFM measurements to check coating uniformity and roughness; material composition and reflectivity were not measured until after production was completed, too late in the mission timeline to address the issue via fabrication modifications.

Despite these shortcomings, however, the tREXS gratings still represent advancements in high-volume, large-area X-ray grating production. The >100 cm$^2$ grating footprint and alignment into modules of 38 gratings per instrument channel demonstrate increased capabilities from previous works (e.g. [15]), and the $\approx$50% absolute diffraction efficiency over the measured band compares favorably to alternative X-ray grating technologies (e.g. [10]). The gratings enable tREXS’ science objectives and were implemented into a multi-channel X-ray spectrograph as described in [13]. Further, the >190 gratings generated from two master gratings with SCIL are the most large-scale grating replicas manufactured with this approach, helping to continue to develop the processes that will allow for higher-volume replication in future applications. Moving forward, the lessons learned in this work can be applied to generate additional gratings with more refined processing and better diffraction performance.

## Data Availability

No datasets were generated or analysed during the current study.
